# Design and methods for a randomized clinical trial of a diabetes self-management intervention for low-Income Latinos: *Latinos en Control*

**DOI:** 10.1186/1471-2288-9-81

**Published:** 2009-12-09

**Authors:** Milagros C Rosal, Mary Jo White, Angela Restrepo, Barbara Olendzki, Jeffrey Scavron, Elise Sinagra, Ira S Ockene, Michael Thompson, Stephenie C Lemon, Lucy M Candib, George Reed

**Affiliations:** 1 Department of Medicine, Division of Preventive and Behavioral Medicine, University of Massachusetts Medical School, 55 Lake Avenue North, Worcester, MA 01655, USA; 2 Department of Medicine, Division of Endocrinology, University of Massachusetts Medical School, 55 Lake Avenue North, Worcester, MA 01655, USA; 3 Brightwood Community Health Center/Baystate Medical Center, 380 Plainfield Street, Springfield, MA 01107, USA; 4 Department of Medicine, Division of Cardiovascular Medicine, University of Massachusetts Medical School, 55 Lake Avenue North, Worcester, MA 01655, USA; 5 Family Health Services of Worcester, MA, 26 Queen Street, Worcester, MA 01610, USA; 6 Rockingham Memorial Hospital, 100 East Grace Street, Harrisonburg, VA 22801, USA

## Abstract

**Background:**

US Latinos have greater prevalence of type 2 diabetes (diabetes), uncontrolled diabetes and diabetes co-morbidities compared to non-Latino Whites. They also have lower literacy levels and are more likely to live in poverty. Interventions are needed to improve diabetes control among low-income Latinos.

**Methods and design:**

This randomized clinical trial tested the efficacy of a culturally- and literacy-tailored diabetes self-management intervention (*Latinos en Control*) on glycemic control among low-income Latinos with diabetes, compared to usual care (control). Participants were recruited from five community health centers (CHCs) in Massachusetts. The theory-based intervention included an intensive phase of 12 weekly sessions and a follow-up maintenance phase of 8 monthly sessions. Assessments occurred at baseline, and at 4 and 12 months. The primary outcome was glycosylated hemoglobin (HbA1c). Secondary outcomes were self-management behaviors, weight, lipids and blood pressure. Additional outcomes included diabetes knowledge, self-efficacy, depression and quality of life. The study was designed for recruitment of 250 participants (estimated 20% dropout rate) to provide 90% power for detecting a 7% or greater change in HbA1c between the intervention and control groups. This is a difference in change of HbA1c of 0.5 to 0.6%.

**Discussion:**

Low-income Latinos bear a great burden of uncontrolled diabetes and are an understudied population. Theory-based interventions that are tailored to the needs of this high-risk population have potential for improving diabetes self-management and reduce health disparities. This article describes the design and methods of a theory driven intervention aimed at addressing this need.

**Trial registration:**

http://www.clinicaltrials.gov # NCT00848315

## Background

Type 2 diabetes (diabetes) is an epidemic in the United States (US) and across the globe [1-5]. Approximately 29 million people in the US alone are expected have a diagnosis of type 2 diabetes in the year 2050, an increase of 165% from the year 2000 [6]. Elderly and low-income and minority individuals and communities are disproportionally affected by diabetes and will experience the most rapid growth in diabetes prevalence [6]. It is estimated that more than 20% of the US Latino population will have diabetes by the year 2030 if current trends continue [7]. In addition to the high burden of diabetes among Latinos, uncontrolled diabetes is prevalent in this population [8-13] and contributes to higher rates of diabetes-related complications and worse overall diabetes outcomes among Latinos compared to non-Latino whites [14,15]. The economic costs of diabetes are staggering and will increase even more with increasing prevalence and the advent of new medical technology [6].

Large efficacy studies have shown that tight glycemic control reduces microvascular complications of diabetes [16-18] and results in related cost savings [19]. However, translation studies to examine how to best implement this knowledge for the purpose of enhancing the health of segments of the population who suffer the greater burden of diabetes, such as low-income Latinos, are sparse [12,20]. Interventions are needed to improve adherence to self-management among low-income Latinos, and in particular Caribbean Latinos, the largest Latino group residing in the northeast US and one of the least studied Latino groups. The purpose of this manuscript is to describe the design and methods of a culturally- and literacy-tailored diabetes self-management intervention (*Latinos en Control*) to improve glycemic control among low-income Caribbean Latinos with diabetes.

## Methods

### Study Design

**Figure 1:**
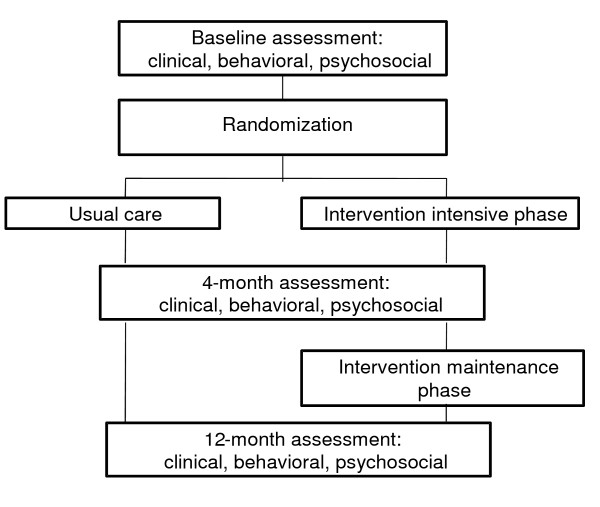
**Study design**.

This study was a prospective randomized clinical trial (RCT), funded by the National Institutes of Health (NIH), that tested the efficacy of a diabetes self-management intervention tailored to the cultural and literacy-needs of low-income Latino patients. The study participants were randomized to the intervention, *Latinos en Control*, or usual care control condition. The duration of the intervention was 12 months and assessments were conducted at baseline, and at 4- and 12 month follow up (see Figure 1 for study design). The primary outcome was glycemic control as measured by reductions in glycosylated hemoglobin (HbA1c) levels. Secondarily, the study sought to:

1) Determine the efficacy of the intervention on diabetes self-management behaviors (i.e., diet, physical activity and self-monitoring of blood glucose (SMBG)), cardiovascular disease risk factors (body mass index (BMI), lipids and blood pressure), and psychosocial factors (including diabetes knowledge, self-efficacy and depressive symptoms);

2) Determine the association between diabetes self-management behaviors (i.e., diet, physical activity and self-monitoring of blood glucose (SMBG)) and the primary endpoint;

3) Evaluate the association of demographic and psychosocial factors with self-management behaviors and HbA1c levels, and with changes in behavior and HbA1c levels;

4) Conduct a qualitative assessment of the intervention components perceived by patients as having the greatest impact on their ability to manage their diabetes; and

5) Determine the cost (per patient) of implementing the intervention.

### Intervention

#### Behavioral targets

Behavioral targets of the intervention included diet, physical activity (PA), blood glucose self-monitoring (SMBG) and medication adherence. Dietary targets included reduction in saturated fat intake while maintaining a balance of mono- and polyunsaturated fats, increased fiber intake through promotion of whole grains, added fiber such as bran and flaxseed, nuts, seeds, and fruits and vegetables, and decreased intake of high glycemic index foods (e.g., starchy vegetables), reduction of sodium intake, and portion control (e.g., rice). Physical activity targets included encouragement to gradually increase walking steps (measured via step counters provided to participants) with an ultimate goal of 10,000 steps per day. Glucose self-monitoring was recommended in the morning prior to breakfast, two hours following a main meal, and any time symptoms of hyper- or hypoglycemia or illness were present. Patients were encouraged to follow the medication regimen prescribed by their providers.

#### Theoretical foundation

Social cognitive theory (SCT) [21], motivational interviewing, the patient-centered counseling model [22-24], adult education principles and practices [25-27] and our previous intervention experiences with this population [28] guided intervention development and implementation. The intervention targeted knowledge gaps and misinformation, promoted positive attitudes toward diabetes self-management (i.e., self-efficacy) and facilitated learning of skills for change in several health behaviors (i.e., diet, PA, SMBG, medication intake) in an effort to increase self-efficacy and behavior change. Strategies used included:

1) Direct instruction and modeling by professionals (i.e., intervention nutritionist or health educator) and peers (i.e., peer models in an educational soap opera video and lay workers from the community that delivered the intervention);

2) Opportunities for mastery experiences through hands-on skill-building activities (e.g., participation in cooking traditional meals utilizing healthy cooking methods; walking and counting steps);

3) Personalized goal-setting, self-monitoring, feedback and problem-solving for building self-management skills; and

4) Activities that facilitated the implementation of targeted skills (e.g., food bingo, making food shopping decisions during a supermarket tour).

As soap operas are popular in the target population and people enjoy and remember their content, a soap opera video was created as a core intervention tool. Through the drama of a Latino woman with diabetes and her daughter with pre-diabetes, the story reinforced key self-management concepts; challenged negative attitudes (i.e., "healthy foods don't taste good"); and portrayed challenges, ambivalence and dilemmas associated with prevention and self-management in a person's daily life (e.g., wishing health for themselves and their family yet cooking unhealthy foods for all). A discussion guide accompanying this video aimed to facilitate the identification of individual values, highlight discrepancies in an individual's values and their behavior, and reinforce the individual's motivations to engage in diabetes self-management and lifestyle change. Group members served as a tool to model, encourage and support successive approximations to optimal self-management behaviors.

#### Intervention format and content

The intervention consisted of an intensive phase (12 weekly sessions) followed by a maintenance phase (8 monthly sessions) for a total duration of one year. The format was primarily group-based, however the first session was conducted individually in the participant's home. All group sessions included a brief coaching segment that included personalized review of progress, problem-solving and new goal-setting, and a safety assessment. Various teams that included professional and trained lay workers implemented the intervention in Spanish.

Each group session included a segment of the above-described soap opera followed by a guided discussion that aimed to emphasize key messages. Accompanying intervention materials aimed to enhance comprehension of complex diabetes self-management information among individuals with varying literacy needs, with emphasis on meeting the needs of very low literate individuals. Materials included a colorful "food guide" book which presented pictures of Latino and other foods categorized by the colors of a traffic light (green, yellow and red) based on saturated or trans-fat content, salt and the glycemic index. A chart similarly colored with the colors of a traffic light pictorially associated ideal, borderline and dangerous glucose levels to attach a meaning to the glucose values (numbers) obtained in self-glucose testing. Low literacy-tailored goal-setting and self-monitoring worksheets accompanied these materials. Intervention participants received a pedometer and instructions for its use along with information on safe places for walking and exercise. Hands-on opportunities for skill development included practice in using healthy cooking methods for ethnic foods at each session, simple instructions on label reading and a supermarket tour that facilitated locating health foods when shopping. Group sessions included culturally popular activities (e.g., watching the soap opera, food bingo games, eating together as a family and, if possible, involving the family) and promoted group cohesion (e.g., ice breaker activities, group sharing and brainstorming), modeling of desirable behaviors and support for change. Group meals accompanied by discussion guides stimulated discussion around taste of the foods prepared, the ease of food preparation, ways of implementing the recipes at home, acceptability to family and friends, and steps to trying new eating styles at home. Guided discussions aimed to reframe pervasive attitudes towards a "diabetic diet" [29] (e.g., "low-fat foods don't taste good"; "the Puerto Rican diet is not amenable to change"; "If I eat only the recommended portions I will be hungry") observed in our previous research. Table 1 presents an outline of the intervention content. Table 2 lists sample strategies and the constructs targeted by each.

**Table 1:** Content of Latinos en Control Intervention.

Session number	Intensive Phase: Session Objectives and Topics.
1	Rapport with individual patients; individual assessments of: DSM history; DSM goals and incentives; expectations and commitment for the program; family support and resources for DSM; rationale for DSM; begin SMBG twice daily.

2	Group cohesiveness (i.e., icebreaking exercises); what is diabetes; meeting and working with a new health care provider; physical activity self-monitoring (step counters); begin walking and physical activity.

3	Attitudes toward healthy eating; healthiest foods ("Green" section of the Traffic Light Food Guide); communicating with dietitians; begin self-monitoring of food intake.

4	Review of "Green" foods; portion control ("Yellow" section of the Traffic Light Food Guide); common challenges to self-monitoring of food intake.

5*	Review dietary concepts introduced up to now; behavior changes made up to now; foods to avoid or eat infrequently and in small amounts ("Red" section of the Traffic Light Food Guide); management of hypoglycemia and self-management; communicating with health care providers *(*Session protocol and materials*)

6	Mid-program review: physical activity, dietary concepts, self-monitoring, understanding and practice of self-management for glucose control, management of hypoglycemia.

7	Medication adherence; cholesterol and blood pressure; diabetes complications; barriers and resources to self-management; foods bingo; what to ask from health care providers.

8	Foot care; infections; smoking; stress management; getting support from the health care system.

9	Food labels and label reading skills; saturated fat, sodium and fiber; food bingo.

10	Supermarket tour.

11	Review food shopping strategies; heart healthy eating; management of sick days; following provider recommendations.

12	Program review; future challenges to maintenance; keeping in touch with health care providers.

	**Maintenance Phase: Session Objectives and Topics.**

13	Review of self-management concepts; continuing to increase physical activity

14	Progress toward healthy eating; new ideas for increasing healthiest foods; continuing to self-monitor self-management behaviors; group problem-solving of challenges.

15	Managing challenges to portion control and avoiding unhealthy foods; Moving more.

16	Review of self-management experiences.

17	Medication adherence; cardiovascular risk factors and diabetes complications.

18	Staying healthy and reducing stress.

19	Future challenges to maintenance of behavior change.

20	Review and graduation.

**Table 2:** Constructs and strategies targeted by the intervention.

	Knowledge	Attitudes	Behaviors			
			**Diet**	**PA**	**SMBG**	**Medication Adherence**

Soap opera with guided group discussion	X	X	X	X	X	X

Group cooking and cooking demonstrations	X	X				

Group meals with guided group discussions	X	X	X			

Multiple presentations of key intervention messages	X		X	X	X	X

Emphasis on one message at a time	X					

Self-monitoring demonstrations	X	X			X	

Cognitive re-framing		X				

Quick quizzes	X					

Modeling		X	X	X	X	

Family support	X	X	X	X	X	X

Behavioral "experiments" (or trials)		X	X	X	X	

Stress management		X	X	X	X	

Label reading	X		X			

Use of measuring aids	X		X	X	X	

Feedback opportunities (logs review, discussion of downloaded BG values, reinforcement of positive attitudes and behaviors)	X	X	X	X	X	X

Visual aids (large visuals, pictorial log sheets, pictorial food books	X		X		X	

Supermarket tour	X		X			

Step counters		X		X		

Goal setting (group and individual)			X	X	X	X

Problem-solving	X	X	X	X	X	X

Group "games"	X	X	X	X	X	X

During the group sessions, each participant spent approximately 10 minutes in a one-on-one discussion with an interventionist. Interventionists used a patient-centered counseling approach, to set behavioral goals, assess progress, provide feedback and facilitate improvements. Interventionists downloaded data from the participants' glucose monitors at each session and used these data in conjunction with the participants' daily logs of SMBG values, dietary intake and physical activity (steps) to provide accurate feedback on blood glucose variability and self-management behaviors.

Significant others (family members or friends living in the participant's household) were invited to attend the group sessions to elicit home-based support for the implementation of the intervention. To promote and support attendance, patients received reminder telephone calls the day before each session and transportation to sessions as needed.

#### Patient safety

A diabetologist oversaw participant safety through review of glucose values downloaded at each session and a checklist of symptoms or conditions utilized by the interventionists at each session. This checklist included questions about any medication changes, urgent or emergency room visits since the previous session attended, new symptoms since the previous session, and foot lesions. It also asked about the steps that the patient followed if a hypoglycemic (<70 mg/dl) or a hyperglycemic (>350) event was observed. Interventionists reviewed safety recommendations with participants who were non-adherent. Following each intervention session, the diabetologist sent an e-mail to primary care providers of participants who had two or more hypoglycemic events, or one hyperglycemic event (>500 mg/dl) since the previous session attended.

#### Intervention fidelity

Several strategies aimed to enhance the fidelity of the intervention delivery. The intervention staff received extensive training in accordance with a systematic protocol which included: 1) diabetes and diabetes self-management, 2) the theoretical framework of the intervention, 3) group management skills, and 3) training in implementing the intervention protocol itself. A behavioral psychologist, two diabetologists, and a clinical research dietitian participated in the training. The training incorporated didactic interactive lessons, mock sessions with feedback, and in-vivo observation followed by debriefing. The initial training for the intervention providers was approximately 40 hours in duration. Intervention delivery was supervised by the psychologist and one of the diabetologists. Fidelity checklists served to monitor delivery or omissions of intervention components. Supervision of interventionists included review of completed checklists following the sessions. Booster training sessions were scheduled quarterly.

### Study Setting

The study team included University of Massachusetts researchers (the study principal investigator, the project manager, diabetologist and biostatistician) and physicians and staff at five community health centers (CHCs) in urban areas of central and western Massachusetts. Table 3 describes characteristics of the participating CHCs. The Institutional Review Boards at the University of Massachusetts Medical School and Baystate Medical Systems approved the study.

**Table 3:** Characteristics of Participating Community Health Centers and Health Services.

Location	Springfield, MA	Worcester, MA	Springfield, MA	Springfield, MA	Worcester, MA
Catchment Area	The North End of Springfield and surrounding areas	Worcester inner city neighborhoods	Springfield inner city neighborhoods	Springfield inner city neighborhoods	Urban housing project (Plumley Village-East)

Total Patient Population	8,000	17,000	6,580	7,000	1,600

% Hispanic	75	50	48	90	65

### Population

Participants were adult Latinos ages 18 and over who were patients at one of the participating CHCs, had a diagnosis of type 2 diabetes documented in the medical chart and a HbA1c level ≥ 7.5 within the prior 7 months, were functionally capable of meeting the activity goals (walking) and had physician approval to participate in the study. Exclusion criteria included inability to understand and provide informed consent (English or Spanish) to participate; a medical condition that precluded adherence to study dietary recommendations (e.g., Crohn's disease, ulcerative colitis, end-stage renal disease); a cognitive/mental (documented dementia; psychiatric hospitalization or suicidality within the prior five years) or physical condition (diagnosis of AIDS or hepatitis C) that precluded participation; no telephone or access to one; plans to move out of the area within the 12-month study period; intermittent use of glucocorticoid therapy within the prior 3 months; acute coronary event (myocardial infarction or unstable angina) within the prior 6 months.

### Participant recruitment

The study liaisons at each CHC, in collaboration with the PI, designed a fact sheet to introduce the study to the primary care providers (PCPs) at each CHC at provider meetings and through e-mails. Liaisons emphasized to the PCPs the importance of the research and the benefits of the potential results to the targeted population and to the CHC. The Study PI also was available to answer questions or address concerns of PCPs. PCPs provided consent for the screening of their patients for study eligibility and for access to their patients' records for the purpose of screening.

The project manager and the PI oversaw the screening and recruitment process by via daily phone meetings and weekly in-person meetings. Bilingual and bicultural Site Research Coordinators (SRCs), were CHC employees hired by the study. The project manager, the software engineer, the study PI and a recruitment and retention consultant participated in training the SRCs in the implementation of the screening and recruitment protocol. An initial pool of adult Latino patients with a diagnosis of type 2 diabetes and a HbA1c test result of 7.5 or greater within the previous 7 months was identified through administrative databases at the CHCs. The SRCs reviewed medical records and completed a scannable form with medical eligibility information (e.g., physical conditions that preclude study participation). The scannable form, identified by study ID only, was sent to the UMass study center for eligibility determination. Eligibility status was then forwarded to the SRCs and recorded in the tracking system. PCPs reviewed their patients found medically eligible by chart review and approved or disapproved their potential participation in the study based on ability to walk or other documented concerns.

Providers signed a letter for approved patients informing them about the study and inviting them to participate in the final eligibility step, a patient interview. This interview assessed information not available in the medical record (e.g., plans to move out of the area, new medical conditions). Approximately two weeks after sending the letter, the SRC contacted patients in person (at the time of CHC visits) or by phone to discuss the study or schedule a time to do so. Eligible and interested patients were invited to enroll the study and complete the informed consent protocol.

The SRCs provided further explanation of the study objectives and demands on participants, and reviewed the study consent forms with interested patients. These included: consent to participate in the study, consent for release of medical records, consent for release of pharmacy refill history, and consent to be photographed or videotaped if randomized to the intervention arm. Enrolled patients were asked to complete baseline assessments prior to randomization.

### Study Measurements

Glasgow's model of diabetes education evaluation [30], guided the measurement approach, emphasizing the assessment of variables in several categories: social/environmental context (e.g. insurance status, family), patient characteristics (e.g., demographics, depression), process and mediating variables (diabetes knowledge, self-efficacy, medical and diabetes history), diabetes self-management behaviors (dietary intake, PA level, SMBG, medication intake), metabolic and physiologic changes (glycosylated hemoglobin), and short- (lipids, BMI) and long-term health outcomes (which were not assessed in this study). The goal of this model is to facilitate answering important questions related to intervention efficacy, including: 1) Was the intervention efficacious? 2) For whom was this intervention most efficacious? and 3) What were the psychosocial and behavioral mechanisms by which metabolic and physiologic and short-term health outcomes were changed [30,31]?

Baseline and 4- and 12-months follow up assessments included several data sources.

1) A clinic-based assessment included measurements of metabolic and physiologic outcomes, recoding of all medications and pharmacy information, and oral administration of a survey with questions on demographics, personal and family history of diabetes, risk behaviors, and perceived health and weight status.

2) Three (2 weekdays and 1 weekend day) unannounced 24-hour recalls of dietary intake [32-36], physical activity [37-39]. and self-monitoring of blood glucose conducted by a trained registered dietitian via telephone interview. Multiple recalls were used to assess day-to-day intra-individual variations in the behaviors of interest [33,34].

3) A psychosocial assessment, also telephone administered, which included measures adapted for the target population [40]. Constructs assessed included depressive symptoms (CES-D [41-43]), knowledge of diabetes and its management (adapted version [44] of the Audit of Diabetes Knowledge [45]), self-efficacy for diabetes self-management (instrument developed for this study [44] modeled after the IMDSES [46,47]), diabetes-specific quality of life (adapted version [40] of the Audit of Diabetes-related Quality of Life [48,49] (Spanish version)), general quality of life (using an instrument modeled after the RAND-12 [50]) and perceived stress (measured by the 4-item Perceived Stress Scale [51]).

4) Co-morbidities and health care utilization were assessed through medical record audits. Participants' diagnostic (ICD9 codes) and health care utilization (inpatient, outpatient and emergency room encounters with corresponding dates, and medical insurance status) data were summarized by trained clinicians for the year prior to study enrollment and the subsequent 12-month period.

5) Process evaluation data came from two sources. Data on participant attendance, adherence to self-monitoring protocols recorded at each session and participants' reported experiences in the study at the 12-month follow up psychosocial interview.

6) Costs associated with the delivery of the intervention, separate from the research activities, were tracked. These costs included: staff training, staff time for session preparation and clean up, staff time for reminder calls and other calls, staff time for session delivery, cost of food used at sessions, space, cost of transportation to the study and intervention materials.

### Training of Study Assessors and Quality Assurance

The training protocol, developed by the study team, was implemented by the project manager, the diabetologists, the study PI, a recruitment and retention consultant, the study dietitian and a physical activity assessment expert. Clinical assessors were individuals with a background as medical assistants or hospital interpreters, the behavioral assessor was a registered dietitian and the psychosocial assessor was a research assistant. All assessors were bilingual and bicultural, and all received training in accordance with a detailed protocol which included instruction on the data collection tools, administration method and documentation process. Assessors also received training in interviewing skills (e.g., rapport building, communication with patients, attention to potential retention problems, strategies to enhance motivation from patients throughout the assessment process). Training sessions included practice sessions and role plays in which the assessor administered the assessment interview under various challenging situations (i.e., difficulties recalling information, interruptions, difficult-to-engage and talkative patients).

The quality control protocol included the review of assessment forms by the project manager who checked for completion. In addition, random assessments were tape-recorded (with participant permission) for the purpose of review and providing feedback to assessors. A trained nutrition reviewer compared nutrition assessment audio tapes to the nutrition software assessment files sent monthly for completeness, and assessed interview technique in the conduct of the interview (e.g., assessor's demeanor and courtesy, leading questions, prompting for complete food descriptions, guidance of the participant for accurate reporting of portion sizes, offering any nutritional advice). Written feedback from this quality control check was provided to the assessor on a timely basis and any concerns were reassessed at the next quality control check.

### Randomization

A stratified randomization scheme was created using Stata's ralloc procedure [52]. The ralloc procedure was used to create balanced sequences of group assignments randomly permuted in blocks of size 2 and 4 within each stratum. The following criteria were used for stratification: CHC site; gender; HbA1c value (<9, ≥9); and insurance (yes, no). A patient was assigned to the appropriate stratum and then assigned to a group based on the random sequence of group assignments. Patients from the same family were assigned to the same study condition. Randomization occurred after the baseline assessment.

### Sample Size

The study was designed for recruitment of 250 participants, estimating a 20% dropout rate that would result in 100 participants per randomized group. This would provide 90% power for detecting (finding a statistically significant difference) a 7% or greater change in HbA1c between randomized groups. This is a difference in change of HbA1c of 0.5 to 0.6%. The estimate was based on preliminary pilot work that estimated the standard deviation of percent change in HbA1c to be 13%. Power estimates assumed a conservative Bonferonni adjustment for 2 comparisons (4 months and 12 months).

### Data management

A screening and recruitment tracking system was developed by the study team for use by the SRCs. This system facilitated tracking the status of each individual in the original patient pool through the screening and recruitment steps, documentation of all contacts between the SRC and patients regarding the study and pre-programmed reports for oversight of screening and recruitment. Additional patients were added to this database at 6 month intervals based on scanning of administrative databases to identify potential new participants.

A separate web-based tracking database was created by a systems engineer and the project coordinator, with input from other members of the research team, to track patients enrolled in the study. Participants were assigned a study identification (ID) number that identified the site as well as the patient. A family ID number also was assigned to participants who had a family member enrolled the study. Upon study enrollment, the study analyst uploaded participant IDs as well as date of birth, gender, insurance status, and contact information onto this system. This system prompted study assessors when assessments were due, allowed documentation of all study contacts and assessments, and allowed for recording of intervention data and uploading of patient glucose meter data.

### Analysis plan for primary and secondary hypotheses

Differences between randomized groups in changes in the primary and secondary outcomes over time (baseline, and 4- and 12-months post-randomization) were estimated and tested using linear mixed models [53]. With multiple time points within individual, the primary model for HbA1c was a linear mixed model that included random intercept and slope terms (time) for each individual:

where the fixed effect terms included I = indicator of intervention, T4 and T12 = indicators of month 4 and month 12, respectively, and the interaction of the intervention and time points (I*T4 and I*T12) which were included to estimate differences in change in HbA1c between randomized groups. A likelihood ratio test comparing nested models (with and without the interaction terms) tested the primary hypothesis of an intervention effect on change in HbA1c.

The same modeling strategy can be used to estimate and test changes in other corollaries of interest (lipids, BP, BMI, depression and QoL) and on mediating variables (dietary intake, physical activity and SMBG; diabetes knowledge, and self-efficacy).

Building on these analyses and using the mixed model framework, models of factors that contribute to or alter the primary outcome, HbA1c, including the effect of potential confounders (e.g., site, patient demographics) can be fit in an effort to provide a best predictor of change in HbA1c and an exploration of the theoretical framework through examination of mediators of the intervention effect.

### Baseline results

Consistent with the recruitment plan, the study recruited a total of 252 participants. Characteristics of the study sample are presented in table 4. Almost half of the sample (46.9%) was under age 55 and the sample was largely female (76.6%), un-married (74.2%), low-literate (56% had 8^th ^grade or less), non-working (88.7%; 61.7% reported to be disable) and poor (50% had annual household incomes lower than $10,000). Most (91.5%) were Puerto Rican and all chose to respond to the assessment interviews in Spanish. Most participants (68.5%) had received a diagnosis of diabetes at least 6 years prior to study participation.

**Table 4:** Description of the study sample (n = 252).

	%
Age	

18-44	17.1%

45-54	29.8%

55-64	32.9%

≥ 65	20.2%

Gender	

Female	76.6%

Male	23.4%

Marital status	

Married or living with partner	25.8%

Divorced/Widowed/Separated	39.0%

Never married	25.2%

Education	

0-4 years	28.6%

5-8 years	27.8%

9-12 years (not HS grad)	19.1%

≥ High-school or GED	24.6%

Employment status	

Working full or part-time	11.3%

Unemployed/looking for a job	3.5%

Disabled	61.7%

Retired	10.9%

Housewife	12.6%

Household income/year	

< 10,000	50.0%

≥ 10,000	50.0%

Country of origin	

Puerto Rico	91.5%

Dominican Republic	4.5%

Other	4.0%

Language chosen for assessment: Spanish	100%

Years since diabetes diagnosis	

1-5	31.5%

6-10	24.5%

11-15	19.1%

16+	24.9%

## Discussion

This is one of few trials testing a theory-based diabetes self-management intervention tailored to low-income Spanish-speaking primarily Caribbean Latinos. As reflected by the sample demographic characteristics, this population has unique challenges to diabetes self-management, which include low literacy, language barriers, poverty and overall low functioning (i.e., high disability). Two large trials with Latinos were conducted previously, one in Texas [12] and one California [20], both of which primarily targeted Mexican Americans. Notable differences exist among various Latino groups which limit the generalizability of interventions and research findings from one group to another. These include not only Spanish language variations, but also differences in region of origin (native land), culture and traditions, migration history, and region of the US where they live. These group differences and living conditions likely impact diabetes self-management among various groups. For example, food traditions and preferences may pose different challenges for change; likewise, differences in climate and weather between the native land and continental US where the group resides may present differences in challenges to physical activity for groups residing in the north, but not the south or southwest. Thus, intervention approaches tailored to specific Latino sub-groups are needed. This trial will provide insights into how to best intervene to improve diabetes self-management among Caribbean Latinos, primarily Puerto Ricans.

The comprehensive intervention used in this study is novel in that it attempted to address the needs of individuals of varying literacy levels, with a special emphasis on meeting the needs of low-literate and illiterate individuals. Novel intervention tools, materials and activities are needed to facilitate health behavior change among individuals that are low-literate and illiterate. If effective, the intervention package will serve as a valuable tool for dissemination.

In conclusion, this study will provide important information about a new approach to improve glycemic control in a high-risk, but largely unstudied, Caribbean Latino population. If efficacious, the intervention will be poised for dissemination. Study products include: a detailed intervention manual, intervention materials, a manual for training of providers, and data on implementation costs.

## Competing interests

The authors declare that they have no competing interests.

## Authors' contributions

MCR, IO, GR, MJW and MT participated in the design of the study; MR, BO and JS participated in the conceptualization of the intervention; MR and ES developed the intervention manual; JS and LC contributed to the recruitment plan; MR, AB and SL participated in measurement development; MR, MW, AR, AB, BO, JS and LC participated in the implementation of the study; and GR is the biostatistician. All authors read and approved the final manuscript.

## Pre-publication history

The pre-publication history for this paper can be accessed here:

http://www.biomedcentral.com/1471-2288/9/81/prepub
